# (4*S*,5*S*)-2,2-Dimethyl-1,3-dioxolane-4,5-dicarbo­nitrile

**DOI:** 10.1107/S1600536813015973

**Published:** 2013-06-15

**Authors:** Alan H. Haines, David L. Hughes

**Affiliations:** a School of Chemistry, University of East Anglia, Norwich NR4 7TJ, England

## Abstract

The title compound, C_7_H_8_N_2_O_2_, formed by dehydration of the corresponding dicarboxamide, crystallizes as rectangular prisms. The mol­ecules have a *C*_2_ axis of symmetry through the C atom bearing the methyl groups and the mid-point of the ring C—C bond, and the 1,3-dioxolane ring adopts the extreme twist conformation of the two possible with this symmetry. This brings the two nitrile groups nearest to a linear arrangement when the mol­ecule is viewed along the ring C—C bond. The correct absolute configuration of the mol­ecule was defined by that of the original starting material, (2*R*,3*R*)-tartaric acid. The packing is largely controlled by a number of C—H⋯N interactions.

## Related literature

For the first syntheses of the title compound, see: Briggs *et al.* (1985 ▶). For determination of the absolute configuration of (+)-tartaric acid, see: Bijvoet *et al.* (1951 ▶). For related structures, see: (4*R*,5*R*)-2,2-dimethyl-1,3-dioxolane-4,5-dicarboxamide, Shainyan *et al.* (2002 ▶); (2*R*,3*S*)-2,3-dihy­droxy-2,3-di­cyano­ethane and (2*R*,3*S*)-2,3-dibenzo­yloxy-2,3-di­cyano­ethane, Rychlewska *et al.* (2008 ▶); and (2*S*,3*S*)-2,3-dibenzo­yloxy-2,3-di­cyano­ethane, Gawroński *et al.* (2007 ▶). For the Flack *x* parameter, see: Flack (1983 ▶).
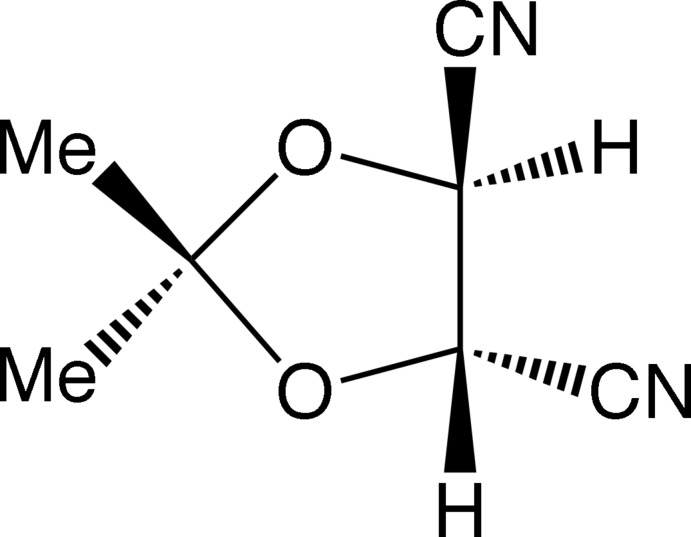


## Experimental

### 

#### Crystal data

C_7_H_8_N_2_O_2_
*M* *_r_* = 152.15
Tetragonal, 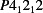

*a* = 8.7740 (2) Å
*c* = 10.0282 (3) Å
*V* = 772.00 (3) Å^3^
*Z* = 4
Mo *K*α radiation
μ = 0.10 mm^−1^
*T* = 140 K
0.08 × 0.07 × 0.07 mm

#### Data collection

Oxford Diffraction Xcalibur 3/Sapphire3 CCD diffractometer
Absorption correction: multi-scan (*CrysAlis PRO RED* RED; Oxford Diffraction, 2010 ▶) *T*_min_ = 0.886, *T*_max_ = 1.000
14922 measured reflections
1133 independent reflections
1073 reflections with *I* > 2σ(*I*)
*R*_int_ = 0.032

#### Refinement

*R*[*F* ^2^ > 2σ(*F* ^2^)] = 0.030
*wR*(*F* ^2^) = 0.076
*S* = 1.09
1133 reflections
67 parameters
All H-atom parameters refined
Δρ_max_ = 0.28 e Å^−3^
Δρ_min_ = −0.11 e Å^−3^

### 

Data collection: *CrysAlis PRO CCD* (Oxford Diffraction, 2010 ▶); cell refinement: *CrysAlis PRO RED* (Oxford Diffraction, 2010 ▶); data reduction: *CrysAlis PRO RED*; program(s) used to solve structure: *SHELXS97* (Sheldrick, 2008 ▶); program(s) used to refine structure: *SHELXL97* (Sheldrick, 2008 ▶); molecular graphics: *ORTEPII* (Johnson, 1976 ▶) and *ORTEP-3 for Windows* (Farrugia, 2012 ▶); software used to prepare material for publication: *SHELXL97* and *WinGX* (Farrugia, 2012 ▶).

## Supplementary Material

Crystal structure: contains datablock(s) I, global. DOI: 10.1107/S1600536813015973/sj5320sup1.cif

Structure factors: contains datablock(s) I. DOI: 10.1107/S1600536813015973/sj5320Isup2.hkl

Supplementary material file. DOI: 10.1107/S1600536813015973/sj5320Isup3.mol

Supplementary material file. DOI: 10.1107/S1600536813015973/sj5320Isup4.cml

Additional supplementary materials:  crystallographic information; 3D view; checkCIF report

## Figures and Tables

**Table 1:** Hydrogen-bond geometry (Å, °)

*D*—H⋯*A*	*D*—H	H⋯*A*	*D*⋯*A*	*D*—H⋯*A*
C2—H2⋯N21^i^	0.946 (13)	2.450 (13)	3.2530 (14)	142.6 (10)

Symmetry code: (i) 
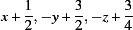
.

**Fig. 1.**
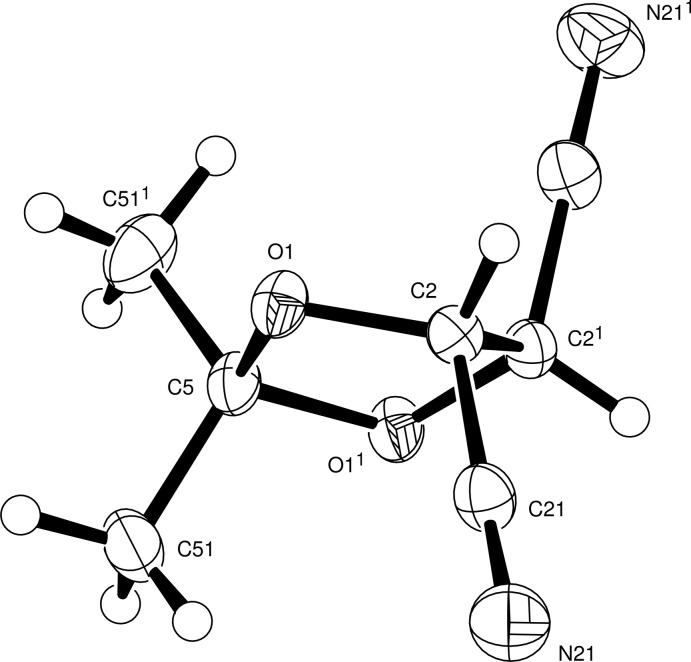
View of a molecule of (4S,5S)-2,2-dimethyl-1,3-dioxolane-4,5-dicarbonitrile indicating the atom numbering scheme. Thermal ellipsoids are drawn at the 50% probability level.

**Fig. 2.**
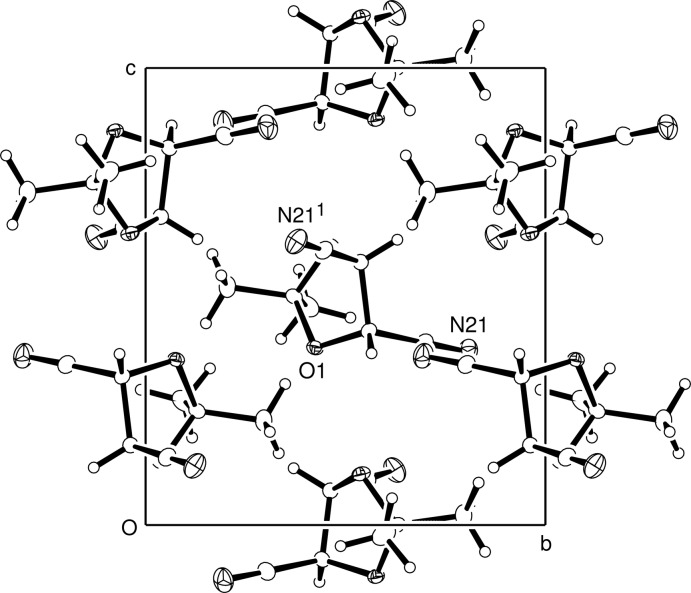
View down the crystallographic a axis showing the apparent alignment of nitrile groups along a twofold screw axis.

## supplementary crystallographic information

### Comment

The stereoisomeric forms of tartaric acid have played a central role in determining the absolute and relative stereochemistries of chiral carbon compounds, and our knowledge of the absolute configurations of such organic compounds stems from determination of the absolute configuration of the sodium rubidium salt of (+)-tartaric acid (Bijvoet *et al.*, 1951). Since that time, many structural determinations by X-ray crystallography have been performed on derivatives of the three isomeric forms of tartaric acid, the chiral (*R*,*R*)- and (*S*,*S*)-isomers and the meso (*R*,*S*)-isomer. Relevant to our structural determination of the title compound are reports on the crystal structures of: (i) its precursor (4*R*,5*R*)-2,2-dimethyl-1,3-dioxolane-4,5-dicarboxamide (Shainyan *et al.*, 2002) - note: the structural diagram in this paper recording the compound's crystallographic data depicts, erroneously, the (4*S*,5*S*)-isomer despite the fact that the stated synthesis is from (2*R*,3*R*)-tartaric acid); (ii) (2*R*,3*S*)-2,3-dihydroxy-2,3-dicyanoethane (Rychlewska *et al.*, 2008); (iii) (2*S*,3*S*)-2,3-dibenzoyloxy-2,3-dicyanoethane (Gawroński *et al.*, 2007); and (iv) (2*R*,3*S*)-2,3-dibenzoyloxy-2,3-dicyanoethane (Rychlewska *et al.*, 2008).

We previously synthesized (4*S*,5*S*)-2,2-dimethyl-1,3-dioxolane-4,5-dicarbonitrile in 80.5% yield as a highly crystalline solid m.p. 163–164 °C by treatment of (4*R*,5*R*)-2,2-dimethyl-1,3-dioxolane-4,5-dicarboxamide with benzenesulfonyl chloride in pyridine (Briggs *et al.*, 1985). [N.B. In the paper by Briggs *et al.*, 1985, the stereochemical descriptors for positions 4 and 5 of the dioxolane ring were incorrectly assigned as *R*; it should be noted that conversion of an amide function into nitrile lowers the order of preference according to the sequence-rules.] Two noteworthy properties of the dicarbonitrile were (i) its resistance to hydrolysis by trifluoroacetic acid-water, an acidic medium which normally brings about ready hydrolysis of the type of acetal group present in this compound, and (ii) the lack of absorptions attributable to a nitrile group in its IR spectrum although an expected absorption was present in its Raman spectrum. Interestingly, (*S*,*S*)-2,3-dihydroxy-2,3-dicyanoethane, which was the desired hydrolysis product of the dicarbonitrile, also proved difficult to synthesize from the unprotected (*R*,*R*)-tartaric acid diamide, was only obtained in low yield, and is not stable at room temperature (Rychlewska *et al.*, 2008). In contrast, (*R*,*S*)-1,2-dihydroxy-1,2-dicyanoethane has been isolated as a stable crystalline solid from the mixture of (*R*,*R*), (*S*,*S*) and (*R*,*S*)-dinitriles obtained in the reaction of glyoxal with potassium cyanide and hydrochloric acid (Rychlewska *et al.*, 2008).

Our molecule has a 5-membered ring with a twist conformation, Figure 1. It lies about a twofold symmetry axis though the Me_2_-carbon atom C5 and the mid-point of the ring C—C bond. Of the two possible extreme twist conformations which may be so defined that one is adopted which brings the two nitrile groups nearest to linearity with the torsional angle C21—C2—C2^1^—C21^1^ at -155.70 (11)°; the alternative twist conformation would set the nitrile groups with a torsional angle near -90°.

When viewed down the crystallographic *a* or *b* axis (Figure 2), intermolecular linear alignment between opposed dipoles of the nitrile groups in neighbouring molecules appears to be a feature of the macro structure of the crystal and might be an attractive explanation for the lack of a nitrile absorption in the IR spectrum of the compound. Thus, dipole moment changes in two parallel (or near parallel) CN groups arranged pointing in opposite directions might "cancel out" leading to a very weak or absent overall absorption for the CN groups. However, the nitrile groups in our structure are not parallel but related by a twofold screw (2_1_) symmetry axis, and the angle between two C–N vectors is 64.35 (8)°.

The absolute configuration of the molecule cannot be determined unequivocally from the X-ray data, but is known from that of the precursor, (2*R*,3*R*)-tartaric acid, and is that shown in Figure 1. We note that the Flack *x* parameter (Flack, 1983) is 0.8 (11), the value of which suggests that we should invert the structure; however, the large s.d. on this value indicates that this parameter has not been reliably determined from the diffraction data.

In contrast to the *C*_2_ symmetry possessed by the dinitrile, the dicarboxamide precursor lacks similar symmetry and the shape of its 1,3-dioxolane ring lies close to an envelope conformation with one of the ring O atoms, O2 in the original publication (Shainyan *et al.*, 2002), 0.503 Å out of the mean plane of the remaining ring atoms; extensive inter- and intra-molecular hydrogen bonding occurs involving the amide groups. In our structure, the packing is largely controlled by a number of C–H···N contacts.

The parent (*S,S*)-1,2-dihydroxy-1,2-dicyanoethane is non-crystalline but the crystal structure of the corresponding (*R,S*)-1,2-dihydroxy-1,2-dicyanoethane has been reported (Rychlewska *et al.*, 2008) and it has a perfectly staggered conformation about the central C—C bond suggesting that an anti-parallel arrangement of vicinal nitrile groups may be a strong driving force influencing conformational preference and thus influencing the choice of twist conformations in the title compound.

### Experimental

The preparation of the title compound, m.p. 163–164 °C (sublimes above 120 °C), [α]_D_ -83 (*c*, 0.6), together with its IR, Raman, and ^1^H and ^13^C NMR spectra has been described (Briggs *et al.*, 1985).

### Refinement

The non-hydrogen atoms were refined with anisotropic thermal parameters. All the hydrogen atoms were located in a difference map and were refined freely.

### Crystal data

**Table:**

C_7_H_8_N_2_O_2_	*D*_x_ = 1.309 Mg m^−^^3^
*M**_r_* = 152.15	Mo *K*α radiation, λ = 0.71073 Å
Tetragonal, *P*4_1_2_1_2	Cell parameters from 5971 reflections
Hall symbol: P 4abw 2nw	θ = 3.1–32.7°
*a* = 8.7740 (2) Å	µ = 0.10 mm^−^^1^
*c* = 10.0282 (3) Å	*T* = 140 K
*V* = 772.00 (3) Å^3^	Cube, colourless
*Z* = 4	0.08 × 0.07 × 0.07 mm
*F*(000) = 320	

### Data collection

**Table:**

Oxford Diffraction Xcalibur 3/Sapphire3 CCD diffractometer	1133 independent reflections
Radiation source: Enhance (Mo) X-ray Source	1073 reflections with *I* > 2σ(*I*)
Graphite monochromator	*R*_int_ = 0.032
Detector resolution: 16.0050 pixels mm^-1^	θ_max_ = 30.0°, θ_min_ = 3.1°
Thin–slice φ and ω scans	*h* = −12→12
Absorption correction: multi-scan (*CrysAlis PRO* RED; Oxford Diffraction, 2010)	*k* = −12→12
*T*_min_ = 0.886, *T*_max_ = 1.000	*l* = −14→14
14922 measured reflections	

### Refinement

**Table:**

Refinement on *F*^2^	Primary atom site location: structure-invariant direct methods
Least-squares matrix: full	Secondary atom site location: difference Fourier map
*R*[*F*^2^ > 2σ(*F*^2^)] = 0.030	Hydrogen site location: difference Fourier map
*wR*(*F*^2^) = 0.076	All H-atom parameters refined
*S* = 1.09	*w* = 1/[σ^2^(*F*_o_^2^) + (0.0398*P*)^2^ + 0.0831*P*] where *P* = (*F*_o_^2^ + 2*F*_c_^2^)/3
1133 reflections	(Δ/σ)_max_ < 0.001
67 parameters	Δρ_max_ = 0.28 e Å^−^^3^
0 restraints	Δρ_min_ = −0.11 e Å^−^^3^

### Special details

**Table:**

Geometry. All s.u.'s (except the s.u. in the dihedral angle between two l.s. planes) are estimated using the full covariance matrix. The cell s.u.'s are taken into account individually in the estimation of s.u.'s in distances, angles and torsion angles; correlations between s.u.'s in cell parameters are only used when they are defined by crystal symmetry. An approximate (isotropic) treatment of cell s.u.'s is used for estimating s.u.'s involving l.s. planes.
Refinement. Refinement of *F*^2^ against ALL reflections. The weighted *R*-factor *wR* and goodness of fit *S* are based on *F*^2^, conventional *R*-factors *R* are based on *F*, with *F* set to zero for negative *F*^2^. The threshold expression of *F*^2^ > 2σ(*F*^2^) is used only for calculating *R*-factors(gt) *etc*. and is not relevant to the choice of reflections for refinement. *R*-factors based on *F*^2^ are statistically about twice as large as those based on *F*, and *R*- factors based on ALL data will be even larger.

### Fractional atomic coordinates and isotropic or equivalent isotropic displacement parameters (Å^2^)

**Table:**

	*x*	*y*	*z*	*U*_iso_*/*U*_eq_	
O1	0.46094 (8)	0.42363 (7)	0.38782 (6)	0.02318 (17)	
C2	0.53777 (10)	0.55849 (10)	0.42482 (9)	0.02062 (18)	
C21	0.44612 (12)	0.69757 (11)	0.39872 (10)	0.0267 (2)	
N21	0.37695 (12)	0.80532 (11)	0.37960 (11)	0.0416 (3)	
C5	0.37130 (10)	0.37130 (10)	0.5000	0.0224 (3)	
C51	0.20468 (13)	0.40426 (15)	0.47877 (13)	0.0359 (3)	
H2	0.6296 (15)	0.5652 (14)	0.3757 (12)	0.026 (3)*	
H51A	0.1892 (16)	0.5121 (18)	0.4539 (15)	0.045 (4)*	
H51B	0.151 (2)	0.3837 (19)	0.5586 (17)	0.052 (4)*	
H51C	0.1670 (19)	0.339 (2)	0.4073 (19)	0.057 (5)*	

### Atomic displacement parameters (Å^2^)

**Table:**

	*U*^11^	*U*^22^	*U*^33^	*U*^12^	*U*^13^	*U*^23^
O1	0.0289 (3)	0.0237 (3)	0.0169 (3)	−0.0052 (3)	0.0036 (2)	−0.0023 (2)
C2	0.0204 (4)	0.0208 (4)	0.0206 (4)	−0.0010 (3)	0.0000 (3)	0.0025 (3)
C21	0.0264 (5)	0.0264 (4)	0.0272 (4)	−0.0018 (4)	−0.0049 (4)	0.0038 (4)
N21	0.0405 (6)	0.0330 (5)	0.0513 (7)	0.0068 (4)	−0.0120 (5)	0.0053 (5)
C5	0.0237 (4)	0.0237 (4)	0.0196 (6)	−0.0054 (5)	0.0034 (3)	−0.0034 (3)
C51	0.0230 (5)	0.0461 (7)	0.0385 (6)	−0.0066 (5)	−0.0005 (4)	−0.0117 (5)

### Geometric parameters (Å, º)

**Table:**

O1—C2	1.4114 (10)	C5—O1^i^	1.4474 (10)
O1—C5	1.4474 (10)	C5—C51^i^	1.5054 (13)
C2—C21	1.4846 (13)	C5—C51	1.5054 (13)
C2—C2^i^	1.5297 (17)	C51—H51A	0.988 (15)
C2—H2	0.946 (13)	C51—H51B	0.946 (18)
C21—N21	1.1397 (13)	C51—H51C	0.975 (19)
			
C2—O1—C5	108.73 (7)	O1—C5—C51^i^	108.28 (5)
O1—C2—C21	112.59 (7)	O1^i^—C5—C51	108.28 (5)
O1—C2—C2^i^	102.49 (5)	O1—C5—C51	110.91 (6)
C21—C2—C2^i^	109.62 (9)	C51^i^—C5—C51	113.15 (13)
O1—C2—H2	108.8 (7)	C5—C51—H51A	110.7 (9)
C21—C2—H2	108.6 (7)	C5—C51—H51B	109.1 (10)
C2^i^—C2—H2	114.8 (8)	H51A—C51—H51B	109.1 (12)
N21—C21—C2	179.18 (12)	C5—C51—H51C	108.7 (9)
O1^i^—C5—O1	105.03 (10)	H51A—C51—H51C	109.3 (15)
O1^i^—C5—C51^i^	110.91 (6)	H51B—C51—H51C	109.9 (14)
			
C5—O1—C2—C21	−88.69 (8)	C2—O1—C5—C51	104.75 (9)
C5—O1—C2—C2^i^	28.99 (9)	O1—C2—C2^i^—O1^i^	−35.25 (11)
C2—O1—C5—O1^i^	−12.00 (4)	O1—C2—C2^i^—C21^i^	84.52 (7)
C2—O1—C5—C51^i^	−130.54 (9)	C21—C2—C2^i^—C21^i^	−155.70 (11)

Symmetry code: (i) *y*, *x*, −*z*+1.

### Hydrogen-bond geometry (Å, º)

**Table:**

*D*—H···*A*	*D*—H	H···*A*	*D*···*A*	*D*—H···*A*
C2—H2···N21^ii^	0.946 (13)	2.450 (13)	3.2530 (14)	142.6 (10)

Symmetry code: (ii) *x*+1/2, −*y*+3/2, −*z*+3/4.
